# The impact of the endocrine-disrupting chemicals on the glucose-6-phosphate dehydrogenase enzyme activity

**DOI:** 10.3389/fphar.2023.1133741

**Published:** 2023-03-13

**Authors:** Duygu Aydemir, Nuriye Nuray Ulusu

**Affiliations:** ^1^ Department of Medical Biochemistry, School of Medicine, Koc University, Sariyer, Istanbul, Turkey; ^2^ Koç University Research Center for Translational Medicine (KUTTAM), Sariyer, Istanbul, Turkey

**Keywords:** G6PD, endocrine disruptors, enzyme, environmental pollutant, oxidative stress

## Introduction

Glucose-6-phosphate dehydrogenase (G6PD, glucose 6-phosphate: NADP (+) oxidoreductase, EC1.1.1.1.49) enzyme is the first and rate-limiting step in the pentose phosphate pathway (PPP). G6PD is an allosteric enzyme found in monomer, homodimer, and homotetramer forms and catalyzes the production of 6-phosphogluconolactone from glucose-6-phosphate (G6P) (Meng et al., 2022). G6PD maintains the reduction of NADP^+^ into coenzyme NADPH which is vital for various reductive biosynthetic reactions such as maintaining reduced glutathione (GSH), protection against reactive oxygen species (ROS), and detoxification of xenobiotics (B Tandogan, 2011; Gao et al., 2019). NADPH is used by the glutathione reductase (GR) enzyme to convert oxidized glutathione (GSSG) into reduced glutathione (GSH) which is one of the most powerful antioxidant molecules in the cell. GSH is used by glutathione s-transferase (GST) enzyme to detoxify xenobiotics such as environmental pollutants, drugs, and chemicals used in industrial products (Aydemir et al., 2018, 2019a; 2019b, 2020a).

G6PD activity is tightly regulated *via* NADPH/NADP^+^ ratio, extracellular oxidants, and posttranslational modifications such as phosphorylation, acetylation, glycosylation, ubiquitinylation, and glutarylation (Aydemir and Ulusu, 2020a). G6PD is the common player in glycolysis, gluconeogenesis, PPP, and lipid metabolism; also, overexpression of G6PD activity is associated with lipid dysregulation, insulin resistance, increased body weight, and obesity (Park et al., 2005). The increasing incidence of diabetes and obesity is the leading cause of death, and disabilities worldwide are considered a major public health problem (Lin et al., 2020). Excessive exposure to industrial products and environmental pollutants such as processed food and beverages is directly associated with an increased risk of obesity, metabolic disorders, and diabetes (Bhupathiraju and Hu, 2016). A wide variety of synthetic chemicals used in industrial products such as cosmetics, pharmaceuticals, food and beverage packaging, dyes, households, pesticides, and hygiene products interfere with hormone metabolism altering the endocrine system in humans and wildlife; thus, they are called endocrine-disrupting chemicals (EDCs) (Metcalfe et al., 2022).

Since EDCs activate peroxisome proliferating activating receptors (PPARs) and exert adverse effects on hormone metabolism, they dysregulate lipid, glucose, and energy metabolisms and are referred to as obesogens (Darbre, 2017). People are exposed to EDCs daily at various concentrations *via* inhalation, digestion, and dermal route. After exposure, these chemicals are metabolized by the liver, kidney, gut, and skin esterases; however, some parts accumulate in the body without metabolizing over time (Aydemir et al., 2020). On the other hand, administration of phthalate and butylparaben altered G6PD activity in various tissues and cells, including the liver, kidney, testis, brain, breast, and spleen, according to the literature (Table 1) (Aydemir et al., 2018, 2019b; 2019a, 2020a). Therefore, we suggest that EDCs can adversely affect the G6PD activity associated with increased body weight, obesity, metabolic syndrome, and diabetes.

**TABLE 1:** Impact of the natural and environmental endocrine disrupting chemicals (EDCS) on the G6PD enzyme activity in different species.

Endocrine Disrupting Chemicals (EDCs)	G6PD activity	Species	References
			
**Natural EDCs**			
			
**Dehydroepiandrosterone (DHEA)**	**↓** Breast cancer cells	Human	Song et al. (2022)
**17 β-estradiol**	**↑** Breast cancer cells	Human	Shin and Koo (2021)
**Genistein**	**↑** Testis	Mice	Godschalk et al. (2022)
**Ferula assafoetida**	**↓** Uterus	Rat	Keshri et al. (2004)
**Melia azedarach**	**↓** Uterus	Rat	Keshri et al. (2004)
**Saraca asoca (Roxb.) de Wilde**	**↓** Blood	Rat	Swar et al. (2017)
**Epicatechin**	**↓** Pure Enzyme	Leuconostoc mesenteroides	Camara et al. (2016)
**Epigallocatechin**	**↓** Pure Enzyme	Leuconostoc mesenteroides	Camara et al. (2016)
**Epicatechin gallate**	**↓** Pure Enzyme	Leuconostoc mesenteroides	Camara et al. (2016)
**Epigallocatechin**	**↓** Pure Enzyme	Leuconostoc mesenteroides	Camara et al. (2016)
**Testosterone**	**↑** Muscle	Rat	Max and Knudsen (1980)
**Estradiol**	**↑** Muscle	Rat	Max and Knudsen (1980)
			
**Environmental EDCs**			
			
**Butylparaben**	**↓** Liver, **↑** Kidney, **↑** Spleen	Rat	Aydemir et al. (2019)
**DEHP**	**↑** Liver, **↑↓** Kidney	Rat	Aydemir et al. (2018)
**Methoxychlor**	**↑** Uterus	Mice	Ghosh et al. (1999)
**Bisphenol A (BPA)**	**↑** Breast cancer cells	Human	Kim et al. (2003)
**4-nonylphenol (NP)**	**↑** Breast cancer cells	Human	Kim et al. (2003)
**4-octylphenol (OP)**	**↑** Breast cancer cells	Human	Kim et al. (2003)
**Zinc**	**↓** Kidney	Lamb	Tandogan and Ulusu (2006)
**Cadmium**	**↓** Kidney	Lamb	Tandogan and Ulusu (2006)

### Impact of EDCs on the G6PD activity *via* hormonal regulation

EDCs can be natural or environmental, affecting synthesis, uptake, or hormone-release mechanism in humans and wildlife (Autrup et al., 2020). Natural EDCs can be classified as phytoestrogens and mycoestrogens. Phytoestrogens are the most prominent group mimicking 17 β-estradiol (E2) hormones that can bind estrogen receptors. Increased G6PD activity has been reported in breast cancer cells upon 17 β-estradiol administrations (Monet JD, 1987; Shin and Koo, 2021). Genistein is found in soybeans as the primary dietary source of phytoestrogens, and it increases the G6PD in transgenic mice with lower G6PD activity (Atm-ΔSRI mice) (Zin et al., 2013; Godschalk et al., 2022).

Ferula assafoetida (“Ferula assa-foetida”) belongs to the Umbelliferae family containing terpenoids and affects the estrogen signaling pathway specifically *via* estrogen receptor α and estrogen receptor β same as phytoestrogens (Ikeda et al., 2002). The extracts of Ferula assafoetida and Chinaberry (“Melia azedarach”) have estrogenic effects, and they cause pregnancy failure when administered to pregnant rats. On the other hand, these extracts exhibit inhibitory effects on the G6PD (Keshri et al., 2004). Saraca asoca (Roxb.) de Wilde, Ashok has estrogenic effects, which is very popular in India; however, dose-dependent G6PD activity increased by using this plant’s extracts, according to the literature (Swar et al., 2017). On the other hand, Epicatechin, epigallocatechin, epicatechin gallate, and epigallocatechin are found in green tea and have anticancer and anti-inflammatory properties. The literature shows they inhibit the G6PD activity (Camara et al., 2016).

G6PD activity is regulated by various hormones (Stanton, 2012), and its activity is considered an indicator of testosterone or estradiol levels. It was proven that injection of the indicated hormones could activate the G6PD activity in a dose-dependent manner (Max and Knudsen, 1980). Xenobiotics, including antibiotics, drugs, and EDCs, can interfere with the secretion, production, metabolism, and transport of estrogen or androgen hormones affecting the G6PD activity. For instance, BPA, dichlorodiphenyltrichloroethane (DDTs), polychlorinated biphenyls (PCBs), and polybrominated diphenyl ethers (PBDEs) have adverse effects on the estrogenic or androgenic pathways associated with reproductive disorders (Amir et al., 2021). Dehydroepiandrosterone (DHEA), androstenolone, is an endogenous steroid hormone precursor. This hormone is produced by the brain, gonads, and adrenal glands and is one of the non-competitive inhibitors of the G6PD in breast cancer cells (Song et al., 2022). Methoxychlor, an insecticide known as an EDC and accepted as a xenoestrogen, can activate the G6PD in mice’s uterus (Ghosh et al., 1999).

### Endocrine-disrupting chemicals can cause a metabolic shift in human and wildlife

EDCs, including bisphenol A (BPA), phthalates, perfluoroalkyl substances (PFAS), and aluminum, contributes to obesity *via* increasing fatty acid storage and appetite (Braun, 2017; Tinkov et al., 2019). EDCs-related obesity may be due to the downregulation of mitochondrial pyruvate carriers (MPC) since, in aerobic conditions, MPC transports pyruvate into mitochondria to be metabolized in the Krebs cycle (Ruiz-Iglesias and Mañes, 2021). Downregulation of MPC results in decreased pyruvate levels in the mitochondria, causing a metabolic shift to the triacylglycerol synthesis associated with obesity (Chen et al., 2018; Hodges et al., 2022). Hypoxia-inducible factor 1α (HIF-α) is one of the essential regulators of the G6PD. EDCs induce HIF-α expression; for instance, BPA can activate HIF-α and vascular endothelial growth factor (VEGF) by triggering the G-protein estrogen receptor (GPER) (Xu et al., 2017; Yang et al., 2021). Both cellular signaling factors, HIF-α and VEGF, can upregulate the G6PD activity (Leopold et al., 2003).

Bisphenols can regulate the nuclear factor erythroid 2–related factor 2 (Nrf2), which controls antioxidant metabolism upon physiological and pathophysiological outcomes of exposure to oxidative stress. Nrf2 transcriptionally induces G6PD activity to upregulate the PPP pathway involved in the antioxidant response of the cell (Salehabadi et al., 2022; H.-C; Yang et al., 2021). Another activator of the G6PD Ataxia-telangiectasia mutated kinase (ATM), a DNA damage-inducible protein kinase that can be activated by BPA (Tichý et al., 2010; Ganesan & Keating, 2016; Xu et al., 2017). 2,3,7,8-Tedtrachlorodibenzo-p-dioxin (TCDD) is one the most toxic EDC with dioxin-like properties inducing TGF-beta1-Smad pathway, oxidative stress, and G6PD activity (Jin et al., 2008; H.-C; Yang et al., 2021). 17beta-estradiol (E2), 4-nonylphenol (NP), and BPA induce the G6PD activity in a concentration-dependent manner in the estrogen-sensitive human breast cancer cell (MCF-7 cells) (Kim et al., 2003).

BPA administration reduces the G6PD activity in erythrocytes leading to hemolysis and morphological changes in the erythrocytes. Thus exposure to EDCs can be life-threatening in G6PD enzyme-deficient individuals (Trivedi et al., 2020). On the other hand, the G6PD was used to explain BPA-mediated diseases in colon cancer cells (SW480), mammary glands, and Sertoli cells because the G6PD was accepted as one of the sensitive biomarkers and can be used for the prediction of BPA-mediated diseases (Ryu et al., 2017). Butylparaben-induced and phthalate-induced oxidative stress *via* altered G6PD activity caused tissue damage in rats’ liver, kidney, brain, and testis tissue (Aydemir et al., 2020).

Toxic metals such as cadmium lead, silver nitrate, thallium sulfate, cobalt, nitrate, arsenic oxide, and arsenic, and essential trace metals copper, nickel, and manganese are found to be related to G6PD enzyme inhibition and various organ damage associated with the pathogenesis of multiple disorders (Tandogan & Ulusu, 2006; 2007). For instance, cadmium and zinc inhibit the G6PD enzyme in lamb kidneys (Tandogan and Ulusu, 2006). On the other hand, exposure to various EDCs can change trace elements’ homeostasis and affect the activity of antioxidant and detoxification enzymes (Akbay et al., 2004; Aydemir et al., 2018; Aydemir, Öztaşcı, et al., 2019; Aydemir & Ulusu, 2020; Anapali et al., 2022). Metals can interfere with the enzymes’ active site, affect the substrate’s binding to the active site and cause reversible or irreversible enzyme inhibitions. Since heavy metals can denature enzymes by disturbing the native state of proteins’ by destroying secondary and tertiary structures’ disulfide bonds, heavy metal ions are accepted as the most effective inhibitors of the G6PD. Workplace exposure to heavy metals such as lead can cause hematotoxicity, hematopoietic malignancies, and mortality in G6PD-deficient individuals (Cocco, 1998; Cocco et al., 2006). G6PD is vital for all living organisms since the detoxification system relies on NADPH production *via* G6PD, 6PGD, and IDH enzymes. On the other hand, individuals with G6PD deficiency are more vulnerable to various diseases, primarily oxidative stress-induced disorders, including endocrine, cardiovascular, and metabolic disorders (Tandogan and Ulusu, 2006; Arese et al., 2012; Aydemir et al., 2018; 2019b; 2019a; 2020; Aydemir and Ulusu, 2020).

## Conclusion

G6PD enzyme is vital for cell proliferation, production of cellular metabolites, cellular energy, oxidative stress status, and antioxidant response in the cell. G6PD is the rate-limiting enzyme of the PPP responsible for NADPH production. NADPH is vital for various reductive biosynthetic reactions, such as maintaining reduced glutathione (GSH), protecting against reactive oxygen species (ROS), and detoxifying xenobiotics, including drugs, environmental pollutants, and environmental pollutants and EDCs. G6PD activity is tightly regulated *via* NADPH/NADP^+^ ratio, extracellular oxidants, and posttranslational modifications such as phosphorylation, acetylation, glycosylation, ubiquitinylation, and glutarylation. Altered G6PD activity is associated with lipid dysregulation, insulin resistance, increased body weight, and obesity since G6PD is the common player in glycolysis, gluconeogenesis, lipid metabolism PPP, and oxidative stress. EDCs are found in most industrial products, including cosmetics, hygiene products, food and beverage packages, toys, and medical devices, and these chemicals interfere with hormone metabolism in humans and wildlife. Since EDCs activate PPARs and adversely affect hormone metabolism, they dysregulate lipid, glucose, and energy metabolisms and are called obesogens. EDCs, including BP, DEHP, BPA, DDT, cadmium, phytoestrogens, PFAS, TCDD, silver nitrate, copper, nickel, manganese, arsenic oxide, and phthalates impair G6PD activity at transcriptional, translational, and posttranslational levels in human and wildlife. Therefore, we suggest that EDCs dysregulate oxidative stress, lipid, and glucose by interfering with G6PD enzyme activity. Individuals with G6PD deficiency can be vulnerable to EDCs-induced adverse health effects, including endocrine, metabolic, and cardiovascular disorders.
